# Disseminating evidence in medical education: journal club as a virtual community of practice

**DOI:** 10.1186/s12909-023-04550-4

**Published:** 2023-08-12

**Authors:** Jonathan Gold, Amit Pahwa, Karen L. Forbes

**Affiliations:** 1 Department of Pediatrics and Human Development, Michigan State University, B215 Clinical Center, 788 Service Road, East Lansing, MI 48824 USA; 2 Department of Pediatrics, Department of Medicine, Johns Hopkins University School of Medicine, Baltimore, MD USA; 3 Department of Pediatrics, University of Alberta, Edmonton, AB Canada

**Keywords:** Undergraduate medical education, Evidence-based practice, Community of practice

## Abstract

**Background:**

This study explores the impacts of the Council on Medical Student Education in Pediatrics (COMSEP) Journal Club, a unique means of providing monthly professional development for a large international community of pediatric undergraduate medical educators. In particular, we sought to establish member engagement with the Journal Club, identify factors impacting member contributions to the Journal Club, and determine perceived benefits of and barriers to participation as a Journal Club reviewer.

**Methods:**

Using an established Annual Survey as a study instrument, six survey questions were distributed to members of COMSEP. Items were pilot tested prior to inclusion. Quantitative data were analyzed using descriptive statistics and chi-square analysis..

**Results:**

Of 125 respondents who completed the survey, 38% reported reading the Journal Club most months or always. Level of engagement varied. Reasons for reading included a topic of interest, keeping up to date on medical education literature, gaining practical tips for teaching and implementing new curricula. Motivators for writing a review included keeping up to date, contributing to a professional organization, and developing skill in analyzing medical education literature, with a minority citing reasons of enhancing their educational portfolio or academic promotion. The most commonly cited barriers were lack of time and lack of confidence or training in ability to analyze medical education literature.

**Conclusion:**

As a strategy to disseminate the latest evidence in medical education to its membership, the COMSEP Journal Club is effective. Its format is ideally suited for busy educators and may help in members’ professional development and in the development of a community of practice.

**Supplementary Information:**

The online version contains supplementary material available at 10.1186/s12909-023-04550-4.

## Background

Staying current with the medical literature can take more time than busy clinicians are able to afford. It is equally important for those involved with health professions education (HPE) to stay abreast of the literature in the HPE field, to ensure their teaching and assessment practices are also evidence based [1]. Although institutions are placing increasing value on formal programs for the development of clinician educators, professional development in this domain does not end with completion of formal programs; clinician educators must engage in the life-long practice of professional development.

Although journal clubs have been used in varying formats as a means of faculty development by regularly reviewing the academic literature in the clinical domain, the medical education literature may be underutilized as a tool to enhance educator development. While smaller institutional journal clubs have been described, [2–4] including synchronous online delivery [5], their use by larger professional organizations has not been reported. Such organizations may conduct discussions of medical education evidence primarily at annual meetings. However, there is a need to engage educators and support their professional development in ways other than infrequent in-person annual meetings. Alternative strategies that reach a bigger audience, such as more frequent, time efficient initiatives, are needed.

Others have considered the education of physicians in general [6], and journal clubs in particular [7], through the lens of *communities of practice*. The theory of community of practice holds that learning does not occur simply through acquisition of new knowledge but rather through participation in a group with a common purpose and a mutual enterprise (or practice). Individuals become members of the group by adopting the language, norms, and culture of the group as well as by participating in group activities.

The Council on Medical Student Education in Pediatrics (COMSEP) is an international professional organization of pediatric clinician educators dedicated to pediatric undergraduate medical education. For more than a decade, COMSEP has electronically disseminated a monthly Journal Club that provides brief reviews of articles published in the medical education literature. The impact of this approach, engaging members and targeting a larger community of practice, has not been previously studied. As a unique means of providing regular professional development for a large community of educators, we sought to explore the benefits and challenges of this initiative in promoting educator development with the following objectives: (1) to establish current COMSEP member engagement with and perspectives on the current COMSEP Journal Club format; (2) to identify factors that motivate COMSEP members to read the Journal Club; (3) to establish benefits perceived by COMSEP members who have completed reviews for the Journal Club; and (4) to identify barriers to contributing to the Journal Club, as perceived by COMSEP members.

## Methods

### Context

The COMSEP Journal Club started in its current format in 2011 as a monthly review of the medical education literature for the edification of its members. Reviewers select a recently published article (within the last 2 years) from the medical education literature on a topic felt to be of interest to the COMSEP membership, with a focus on pediatric medical student education. Reviewers then write a brief 300 to 350 -word summary of the article in a standard format, with the following four sections: (1) What was the study question?; (2) How was it done?; (3) What were the results?; (4) What are the implications? (how can this be applied to my work in education). These reviews are edited by the COMSEP Journal Club editors, who provide brief additional commentary to foster readers’ reflection on applicability of the study to their educational contexts. Three to four reviews are curated and published each month. The Journal Club editors oversee the process of recruiting reviewers, assist with and ensure suitability of article selection, edit reviews, and disseminate in electronic format the monthly Journal Club to the general COMSEP membership. An example of a Journal Club review is included as Supplemental File 1.

The COMSEP Journal Club was established to meet the needs of its members in the following ways: (1) to give reviewers experience examining and applying evidence in medical education; (2) to provide the general COMSEP membership a brief synopsis of important and relevant articles in the medical education literature in order to improve the evidence behind their medical education decisions; and (3) to give COMSEP members an opportunity to enhance their educational portfolios by serving as reviewers and giving back to the COMSEP community.

**Fig. 1:**
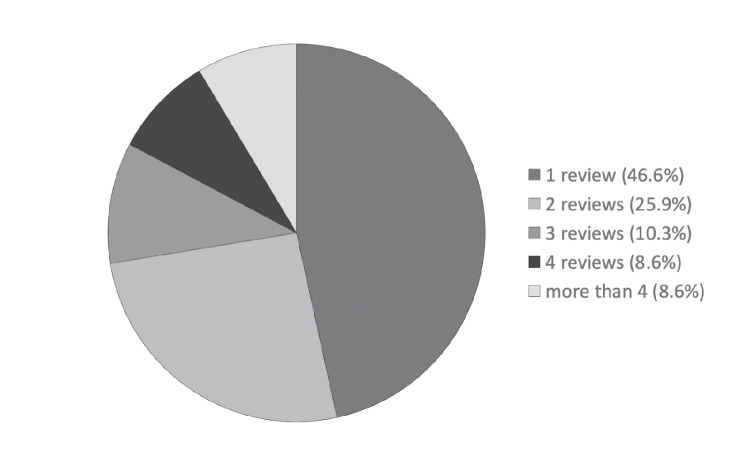
Number of journal club reviews completed by 58 COMSEP member reviewers, over a three-year period

Review of our data on COMSEP members’ contributions to the journal club, along with primary journal sources and review topics, demonstrates that over a three-year period, 58 reviewers contributed a total of 129 article reviews, with the majority of reviewers contributing more than one review (Fig. 1). These reviews came from more than 20 different medical education journals and represented a wide range of themes (Table 1). However, we had no data regarding if and to what degree COMSEP members read the Journal Club article reviews and/or use them for their educational practice, nor regarding the benefits to members, real or perceived, for their contributions to the Journal Club. Specifically, we did not know if members include their reviews in their curriculum vitae or educational portfolios and, if so, whether they proved helpful in the path to promotion. Finally, given challenges with recruitment of COMSEP members to complete reviews, we wished to explore barriers to participation as a reviewer for the Journal Club, in an effort to identify and implement strategies to support members’ contributions. Using a continuous quality improvement lens, we sought to explore these issues so the COMSEP Journal Club could evolve to meet the needs of the membership.

**Table 1:** Examples of medical education journals as source of articles for reviews, and examples of common content areas or themes of articles reviewed

Source of articles for reviews	Article content areas of reviews
*Academic Medicine* *BMC Medical Education* *Medical Education* *Medical Teacher* *Teaching and Learning in Medicine* Others (19)	assessment bias clinical reasoning curriculum development faculty development professional identity formation technology wellness

### Data Collection and Analysis

In the spring of 2021 we surveyed members of COMSEP about their perceptions of COMSEP’s current Journal Club format using the established COMSEP Annual Survey as a study instrument. COMSEP’s membership is composed of leaders in pediatric undergraduate medical education and includes pediatric clerkship directors, site directors, subinternship directors, and other associated faculty and clerkship administrators. Annual survey topic areas are solicited and then chosen by a committee based on their importance to the COMSEP membership and the medical education community at large. Topics are chosen by blinded peer review, and questions are pilot tested prior to inclusion. Questions for each topic area are limited to a response time of a few minutes, to maximize response rate for the overall survey, as per the directive of the COMSEP Annual Survey Committee.

All COMSEP members were invited to participate electronically in the survey via email. Weekly reminders were sent to non-responders for four weeks, at which point the survey closed. Participation was voluntary and confidential.

The complete annual survey consisted of 43 questions, six of which were applicable to the COMSEP Journal Club; the remaining questions related to member demographics and other study topics approved by the survey committee. Our portion of the survey focused on current member participation in the Journal Club both as readers and reviewers, factors that motivate members to read the Journal Club, and perceived benefits and barriers to writing reviews. Specific questions can be found in Supplemental File 2. This study, as related to the Journal Club items, was approved by the Institutional Review Board of the University of Alberta.

Data were analyzed using Microsoft Excel for descriptive statistics (Microsoft Corporation, 2018) and R Core Team for chi square analysis (R Core Team, 2021).

## Results

One hundred twenty-five respondents (27%) completed the annual survey, with 90 (19%) responding to Journal Club survey questions.. Respondents to the annual survey included 35 core clerkship directors (31.5%), 19 associate or assistant clerkship directors (17.1%), and 17 faculty associated with the dean’s office of office of medical education (15.3%). There were 77 women (69.4%). Forty-four percent were assistant professors, 25% were associate professors, and 25% were full professors.

Almost all respondents (92%) indicated they read the Journal Club; thirty-four (38%) respondents reported that they read the Journal Club most months or always and 49 (54%) reported that they read the Journal Club occasionally (a few times a year).

The majority (71.1%) reported that they only read those articles that interested them. Readers’ level of engagement with the process varied with 28 (31.1%) indicating that they skimmed the review for the main points, 44 (49%) reporting that they read the entire review and 14 (15.6%) reporting that they read the review and the article it referenced.

Respondents’ reasons for reading the Journal Club were multiple, and also varied. Respondents could select as many responses as were applicable. Seventy-five respondents (83.3%) reported that they read the reviews if the topic interested them. Seventy-two respondents (80%) read the reviews to keep up to date on the medical education literature. Thirty-eight (42.2%) read to get practical tips on their own teaching and 25 (27.8%) to develop and implement new curricula. Other motivators included developing skill in analyzing the medical literature, to stimulate one’s research ideas, and if a colleague had written a review. Further reasons cited included to share with colleagues who are clerkship directors in other fields, or to share with colleagues who are doing research or developing a project in a related area.

We also asked respondents about the impact of alternative formats on the likelihood that they would engage in the Journal Club. The majority of respondents (60%) reported that a theme issue (focused on a single, specific topic area, such as assessment or professional identity formation, for instance) would make them neither more nor less likely to engage. When it came to the use of virtual technology to enhance the Journal Club experience, a majority (63%) reported that a brief virtual discussion would make them less likely to engage and a substantial minority (40%) reported that they would be less likely to engage in a podcast version of the Journal Club as well.

When comparing the experience of those who had previously written a review with those who had not, we found that reviewers were more likely to report that they read the Journal Club most months (57.9% versus 32.3%), *p* < 0.05) and that they read every review in a given month (47.3% versus 16.1%, *p* < 0.05). Other responses were similar between the two groups.

**Fig. 2:**
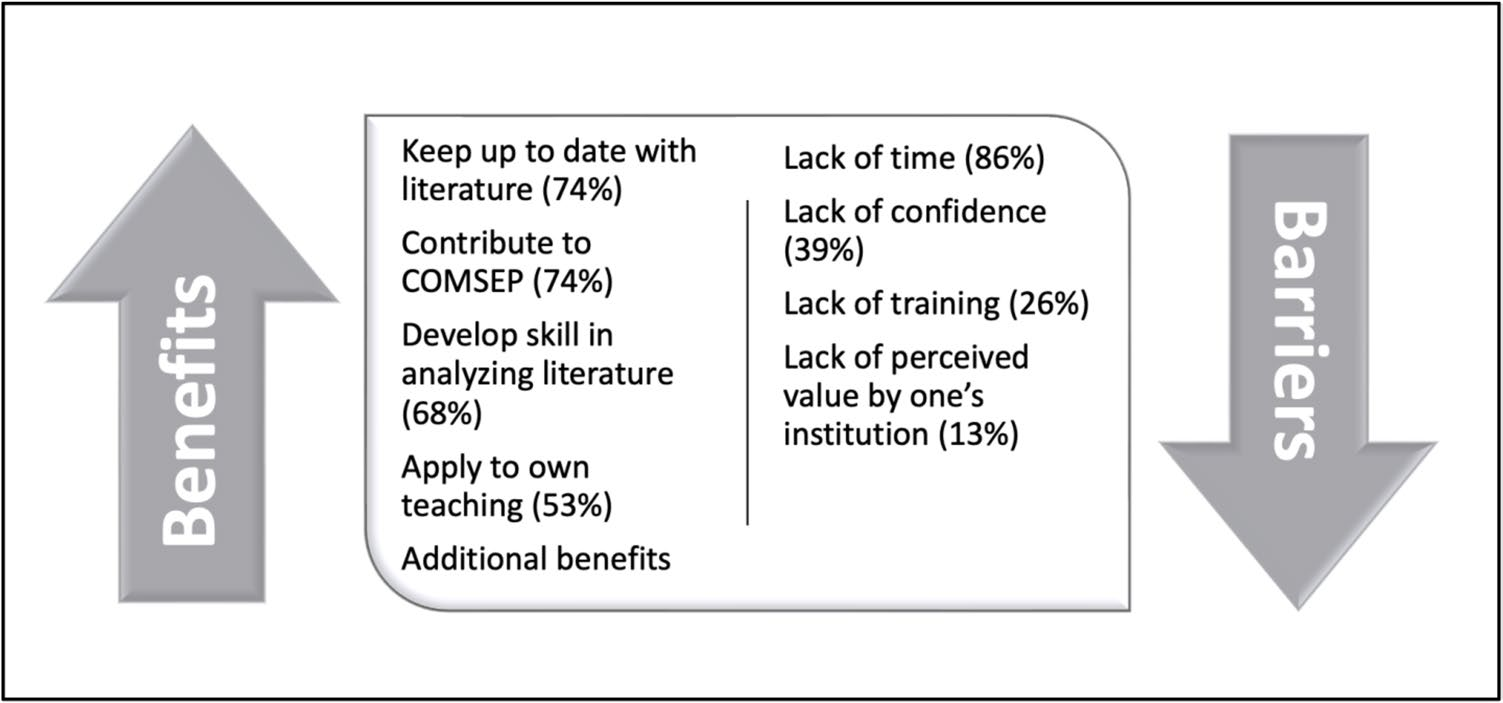
Reviewer perspectives on the benefits and barriers to contributing to the COMSEP Journal Club by writing a review. Additional benefits include addition to one’s curriculum vitae (42%), addition to one’s educational portfolio (32%), to coach trainees in analyzing medical education literature (26%), to simulate one’s own research ideas (26%) and for academic promotion (16%). Respondents could select multiple responses

Nineteen (15%) respondents reported that they had written a review in the past. Among them, there were a wide range of perceived benefits. Fourteen (73.7%) reported that they did so to keep up to date with the medical literature, and the same number reported that they did so to contribute to COMSEP. Thirteen (68.4%) reported that they did so to develop their own skill in reading and analyzing the medical education literature. Ten (52.6%) reported that they did so in order to apply it to their own teaching. A minority of respondents reported benefits such as adding the review to their educational portfolio or curriculum vitae, using the review for academic promotion, coaching of trainees, or stimulating their own research ideas. On the other hand, the most common barrier among all respondents was perceived lack of time to do so (85.6%). Other barriers included lack of confidence in one’s ability to analyze and critique medical education literature (38.9%), lack of training (25.6%) or lack of value by one’s institution or for promotion (13.3%). Those who had contributed reviews were less likely to report that article selection or lack of training were barriers (*p* < 0.05). These perspectives of benefits and barriers to writing a review are depicted in Fig. 2.

## Discussion

The results of this study suggest that as a strategy to disseminate the latest evidence in medical education to its membership, the COMSEP Journal Club is highly effective. The vast majority (92%) of respondents reported that they read the Journal Club at least a few times a year, and more than one third of respondents (38%) read it most months. There are several examples of journal clubs focused on medical education [2–5, 8–12], but none describe the asynchronous virtual format used by COMSEP. This model has the advantage of allowing rapid engagement with a large number of educators. It is also ideally suited to a post-pandemic world in which a large number of educators congregating together carries its own challenges.

Just as with publications in clinical research, there are variable reasons that members choose to read a particular review. Some are looking to address a particular question based on their own needs in teaching or curriculum development, others are perusing the reviews to keep ‘up to date’ in general, while a minority are using the reviews as a springboard for their own research. A recent study suggested that, analogous to the practitioners of clinical medicine, educators prefer synthesized or mediated reviews rather than accessing the primary literature themselves [1]. The format of the COMSEP Journal Club may be ideally suited for busy educators, as each review is limited to 350 words and is organized to allow easy identification of the key points of each article. Further, our results demonstrating that respondents would be less likely to engage in a virtual discussion or podcast as an alternate format suggest that the asynchronous nature of the current format holds appeal to readers due to its accessibility and ability to incorporate into busy schedules.

For reviewers themselves, a majority listed contributing to COMSEP as a large professional organization as a key benefit of reviewing. Many also reported that the activity helped them develop their skills in critical appraisal of the medical education literature. Both reasons suggest that serving as a reviewer creates a sense of belonging to a community of practice, allowing reviewers to both adopt the language of and contribute to the enterprise of undergraduate medical educators. It is not surprising that those who contribute reviews read Journal Club reviews more frequently than non-contributors, and are also less likely to see issues like article selection and training as barriers to writing. Contributing to the project both as consumers and producers likely indicates movement from peripheral to full participation within the community of practice as described by Cruess et al. [6].

Others have made the connection between journal clubs and communities of practice [11, 12], describing the ways in which social learning and professional identity formation come together during these activities. It is interesting to see the same themes at work in an asynchronous online format. In particular, contributing as a reviewer to the Journal Club is aligned with the COMSEP strategic plan, which includes member engagement and professional development [13].

Our study is limited by the response rate and potential biases of those COMSEP members who responded to the Annual Survey and specifically the Journal Club questions; the respondents may reflect those members who are more engaged in COMSEP as an organization and may not reflect the entire membership. Future studies may explore in more detail the way in which engagement with similar formats lead to actual change in educational practice.

## Conclusions

Brief reviews of recent articles in medical education can be effective in engaging clinician-educators both as readers and as authors. Previous studies have examined the use of the journal club format to disseminate evidence about medical education, but this is the first to describe the use of an asynchronous format for a large membership organization.

For others looking to encourage professional development and professional identity formation among a community of medical educators separated both geographically and by institution, the experience of the COMSEP Journal Club might serve as a useful model. The demographics of the respondents suggest that educators in a variety of roles and at different levels of experience find the format engaging.

### Supplementary Information

**Additional file 1.**

**Additional file 2.**

## Data Availability

The datasets used and/or analyzed during the current study are available from the corresponding author on reasonable request.

## Abbreviation

COMSEP: Council on Medical Student Education in Pediatrics
