# Supplementary material for: Disseminating evidence in medical education: journal club as a virtual community of practice
Source: BMC Med Educ. 2023 Aug 12;23:572. doi: 10.1186/s12909-023-04550-4 (PMC10422831; doi:10.1186/s12909-023-04550-4)
Supplement: Supplementary file 1 — Additional file 1. [file 12909_2023_4550_MOESM1_ESM.docx]

**Professionalism --in the Eye of the Beholder**

Tabatabaei ZS, Mirzazadeh A, Amini H, Mafinejad MK. **What We Think About Professional and Unprofessional Behaviors: Differences Between the Perception of Clinical Faculty Members and Medical Students**. BMC Med Educ 2022; 22:866. <https://doi.org/10.1186/s12909-022-03874-x>

Reviewed by XXXXX

**What was the study question?**

What are the perspectives of medical students and faculty about prioritization of elements of professional conduct and how are they different?

**How was this study done?**

A multi-stage qualitative study was conducted at Tehran University of Medical Sciences from 2020-2021. In the first phase, a systematic review of the literature was conducted. This was followed by a consensus method to combine the viewpoints of clinical faculty members and students. As part of the consensus method, participants were asked to produce 5-7 common professional and unprofessional behaviors that were then combined with the codes from the literature search then categorized into six domains: Honor/Integrity, Altruism, Excellence, Justice, Respect, and Responsibility. The resulting behaviors were then prioritized based on importance and compared between clinical faculty and students.

**What were the results?**

Of the 1,547 articles identified, 62 were included for data extraction. From these articles, codes were extracted and classified as professional or unprofessional behaviors. In prioritizing behaviors important to evaluate, clinical faculty ranked unprofessional behaviors over the medical students, who felt that focus should be on professional behaviors. Interestingly, the most important domain of professional and unprofessional behaviors as rated by medical students was Altruism, which was the least important for clinical faculty.

**How can I apply this article to my work in education?**

Clarifying professional and unprofessional behaviors is essential for developing fair evaluation tools. More importantly, having a conversation among clinical faculty and medical students regarding what these behaviors are and addressing conflicting perspectives paves the way for better understanding by both clinical faculty and medical students.

*Editor’s Note: Buried in this somewhat complicated article are other interesting differences in values between students and faculty, among them that students consider paying attention to necessary safety measures for themselves and others a high priority while faculty do not and faculty consider ‘not showing respect and gratitude towards the more experienced faculty members’ more important than students. One thing is clear--the road to professional identity formation is neither smooth nor direct.*
