# Supplementary material for: Disseminating evidence in medical education: journal club as a virtual community of practice
Source: BMC Med Educ. 2023 Aug 12;23:572. doi: 10.1186/s12909-023-04550-4 (PMC10422831; doi:10.1186/s12909-023-04550-4)
Supplement: Supplementary file 2 — Additional file 2. [file 12909_2023_4550_MOESM2_ESM.docx]

**Supplemental File - Survey Questions**

**COMSEP Annual Survey – The COMSEP Journal Club**

The COMSEP Journal Club started in its current format in 2011 as a monthly review of the medical education literature that is distributed electronically for the edification of its members.

These questions will explore how COMSEP members use the Journal Club in its current format for their personal and professional development and educational practice, as well as explore members’ views on the perceived benefits and barriers of contributing to the Journal Club.

1. Please indicate your experience with the current format of the monthly edition of the COMSEP Journal Club:

| I read the COMSEP Journal club: | Never  O | Occasionally (few times/year)  O | Most months or always  O |  |
| --- | --- | --- | --- | --- |
| In a typical month I read: | No reviews  O | Only those reviews that interest me  O | All of the reviews  O |  |
| If I find a review that interests me I usually: | Skim it for the main points  O | Read the entire review  O | Read the review and the article it references  O | Not applicable  O |
| The current length of each review (about 350 words) is: | Too short  O | Just right  O | Too long  O | Not applicable  O |
| The current number of reviews per edition (3-4) is: | Too few  O | Just right  O | Too many  O | Not applicable  O |

1. What motivates you to read a COMSEP Journal Club review? Select up to three (3) reasons.

- The topic of the article is of interest to me
- To develop and implement new curricula in my own course
- To develop skill in reading and analyzing medical education literature
- To get maintenance of certification (MOC) credits
- To get practical tips for my own teaching
- To keep updated in the medical education literature
- To stimulate my own research ideas
- Other(s) (free text) _____________________________________________
- Not applicable

1. Compared to the current journal club format, how likely would you be to engage in the following formats of the journal club?

|  | Less likely than current format | Same as current format | More likely than current format |
| --- | --- | --- | --- |
| A ‘theme’ issue (for example, assessment or professional identity formation) | O | O | O |
| A podcast version of the review | O | O | O |
| Brief scheduled virtual discussion of articles | O | O | O |

Do you have any other suggestions for changes to the current format? (free text) _____________________________________________

1. Regardless of whether or not you have written a review in the past, what barriers do you perceive to preparing a review? Select up to three (3) reasons.

- Article selection
- Confidence in my ability to analyze and critique medical education research
- Lack of time
- Lack of value or recognition by my own institution or for promotion
- No training
- Review format and length
- Unclear expectations
- Other(s) (free text) _____________________________________________

1. Have you written reviews of scholarly articles for the monthly COMSEP Journal Club?

- yes
- no

1. If you have been a reviewer for the COMSEP journal club, what benefits do you perceive? Select all that apply.

- Academic promotion
- Addition to CV
- Applications to my own teaching activities
- Coaching of trainees in analyzing medical education literature
- Develop skill in reading and analyzing medical education literature
- Educational portfolio
- Keep updated in the medical education literature
- Maintenance of certification credits
- Stimulate my own research ideas
- To contribute to COMSEP
- Other(s) (free text) _____________________________________________
